# Discordant Effects of Cannabinoid 2 Receptor Antagonism/Inverse Agonism During Adolescence on Pavlovian and Instrumental Reward Learning in Adult Male Rats

**DOI:** 10.3389/fnsyn.2021.732402

**Published:** 2021-08-30

**Authors:** Danna Ellner, Bryana Hallam, Jude A. Frie, Hayley H. A. Thorpe, Muhammad Shoaib, Hakan Kayir, Bryan W. Jenkins, Jibran Y. Khokhar

**Affiliations:** Department of Biomedical Sciences, University of Guelph, Guelph, ON, Canada

**Keywords:** endocannabinoid, appetitive, autoshaping, conditioning, adolescent

## Abstract

The endocannabinoid system is responsible for regulating a spectrum of physiological activities and plays a critical role in the developing brain. During adolescence, the endocannabinoid system is particularly sensitive to external insults that may change the brain’s developmental trajectory. Cannabinoid receptor type 2 (CB2R) was initially thought to predominantly function in the peripheral nervous system, but more recent studies have implicated its role in the mesolimbic pathway, a network largely attributed to reward circuitry and reward motivated behavior, which undergoes extensive changes during adolescence. It is therefore important to understand how CB2R modulation during adolescence can impact reward-related behaviors in adulthood. In this study, adolescent male rats (postnatal days 28–41) were exposed to a low or high dose of the CB2R antagonist/inverse agonist SR144528 and Pavlovian autoshaping and instrumental conditional behavioral outcomes were measured in adulthood. SR144528-treated rats had significantly slower acquisition of the autoshaping task, seen by less lever pressing behavior over time [*F*_(2, 19)_ = 5.964, *p* = 0.010]. Conversely, there was no effect of adolescent SR144528 exposure on instrumental conditioning. These results suggest that modulation of the CB2R in adolescence differentially impacts reward-learning behaviors in adulthood.

## Introduction

The endocannabinoid system (ECS) is responsible for the regulation of many biological systems through the activities of endogenous cannabinoids (eCB) such as anandamide and 2-arachidonoylglycerol, eCB synthesizing and degrading enzymes, and the endogenous receptors cannabinoid receptor 1 (CB1R) and CB2R. The CB2R had traditionally been labeled a peripheral cannabinoid receptor but its more recent discovery in the brain (Atwood and Mackie, 2010) has garnered interest regarding its role in the CNS. Studies have localized CB2R expression to regions important to the mesocorticolimbic signaling pathway, namely the prefrontal cortex (PFC), nucleus accumbens (NAc), and ventral tegmental area (VTA) (Aracil-Fernandez et al., 2012; Zhang et al., 2014). In particular, CB2R are expressed on the dendrites of dopamine (DA) neurons in the VTA, which project to the NAc and are integral to reward response (Zhang et al., 2017). Activation of the CB2R inhibit DAergic neurons firing, and ultimately decreases DA release into the NAc (Zhang et al., 2014; Han et al., 2017; Galaj and Xi, 2019).

Despite their identification in reward-relevant brain regions, only a handful of studies have explored the relationship between CB2R activation and modulation on related behavioral processes. CB2R activity appears to modulate reward and drug seeking behavior, in line with their expression in the mesocorticolimbic pathway (Zhang et al., 2014; Ghosal et al., 2019; Martin-Sanchez et al., 2019). Inhibition of DAergic neurons projecting from the VTA to the NAc by CB2R activation mitigates cocaine self-administration in adult mice (Zhang et al., 2014). Additionally, CB2R antagonist/inverse agonist exposure during the acquisition phase of conditioned place preference testing decreased in the rewarding effects of alcohol (Martin-Sanchez et al., 2019). These studies suggest that the CB2R plays a direct role in adulthood drug and reward seeking behavior. Importantly, however, it is unclear if CB2R modulation during critical periods of mesocorticolimbic development has any lasting impact on future reward response.

Adolescence is characterized by various developmental changes that occur in the period between childhood and adulthood (Spear, 2000). During this time, there is extensive reorganization of cortical and limbic neurocircuitry, which contributes to natural cognitive, emotional, and reward development (Paus et al., 2008). Many neurotransmitter systems fluctuate significantly during this period of neurodevelopment (Meyer et al., 2018; Thorpe et al., 2020), and make the brain highly susceptible to social, nutritional and environmental influences, as well as insults by drugs of abuse (Thorpe et al., 2020). The ECS has been shown to play a crucial role in adolescent neuronal development, including modulating the ratio of excitatory and inhibitory signaling in the PFC (Meyer et al., 2018), and CB2R expression specifically increases in the PFC, NAc, and hippocampus during the rat equivalent of adolescence (Amancio-Belmont et al., 2017). The existence of adolescent-specific stressors such as family, academic, and peer pressures make this a unique period for environmental modulation of ECS activity, which has been extensively implicated in stress response (Tottenham and Galvan, 2016). In addition, adolescents are especially vulnerable to drug use. Cannabis is one of the most commonly used drugs among this age group, so understanding the impact of adolescent cannabinoid exposure is of utmost importance (Hamidullah et al., 2020, 2021). Previous human and animal studies suggest that adolescent exposure to cannabinoids dysregulates ECS activity, which may have long-term behavioral implications that can persist into adulthood (Hamidullah et al., 2020; Thorpe et al., 2020). For instance, treatment of adolescent rats with the CB1R/CB2R agonist WIN55,212-2 (Schoch et al., 2018) or the CB1R/CB2R partial agonist and the primary psychoactive constituent of cannabis Δ9-tetrahydrocannabidiol (THC) (Kruse et al., 2019) have been shown to modulate the response to food-predictive cues in adulthood.

Since CB2R have been implicated in the rewarding effects of a variety of drugs (e.g., nicotine, alcohol) (Ishiguro et al., 2007; Navarrete et al., 2013), our interest was assessing the persisting impact of adolescent CB2R modulation on future reward learning. Furthermore, since pharmacological modulation of CB1R and CB2R has been shown to impact reward learning in adulthood, we focused on the impact of CB2R modulation on two forms of reward learning behaviors: Pavlovian conditioning and instrumental conditioning. Autoshaping is a form of Pavlovian conditioning in which an animal develops a response to a neutral stimulus (i.e., a lever) that predictive of an outcome, such as the delivery of a palatable food reward, that is not contingent on the animal’s response. Behaviors exhibited during this task are reflective of either sign-tracking or goal-tracking, which are the attribution of incentive salience to the stimulus or the place of reward delivery (i.e., food dispenser), respectively (Flagel et al., 2010). Instrumental learning is a form of operant conditioning, in which the delivery of a reinforcer is contingent on an animal’s behavior, and thus exhibiting said behavior is considered goal-directed. While there is some overlap between the circuitry mediating Pavlovian vs. instrumental conditioning, the exact mechanisms behind these learning behaviors may be unique (Cain and LeDoux, 2008; Guo et al., 2016; Bouton et al., 2021; Doñamayor et al., 2021). Pavlovian and instrumental learning studies both demonstrate ventral striatum activation, with Pavlovian tasks showing higher recruitment of the left putamen of the dorsal striatum than instrumental tasks, which preferentially activate the caudate (Chase et al., 2015).

While existing studies have examined behavioral outcomes linked with short-term modulation of the CB2R (Aracil-Fernandez et al., 2012; Zhang et al., 2014), the long-term effects of CB2R modulation during adolescence remain largely unknown. Thus, the objective of our study was to understand the role of CB2R in adolescent development as it relates to reward learning in adulthood and processing by pharmacologically inhibiting CB2R during adolescence in a rodent model. We hypothesized that adolescent CB2R inhibition would hamper acquisition of reward-paired lever pressing in both the autoshaping and instrumental reward-learning tasks in a dose-dependent manner.

## Materials and Methods

### Subjects

Male Sprague-Dawley rats (*n* = 7–8/experiment/group) were obtained from Charles River Laboratories (Saint Constant, Quebec) at postnatal day (PND) 21. Males were used as previous studies report inconsistent effects of SR144528, the antagonist/inverse agonist used in this study, in females (Craft et al., 2012). All rats were weaned at PND23 and pair-housed on a 12:12 light:dark cycle. Rats were food restricted to 85–90% of their baseline body weights beginning on PND56 to encourage food-motivated behavior and exploration during behavioral testing. Animal care, behavioral testing, anesthesia, and euthanasia procedures were performed in accordance with the Animal Use Protocol approved by the University of Guelph Animal Care Committee.

### Drug Administration

SR144528 is a potent and highly selective CB2R antagonist/inverse agonist (Rinaldi-Carmona et al., 1998; Portier et al., 1999) and was obtained from the National Institute of Mental Health Drug Repository Program. SR144528 was dissolved in a 1:1:18 vehicle solution of Cremophor: 95% ethanol:0.9% saline. Rats were given intraperitoneal injections once daily from PND28 to PND41. Rats received either 0, 3.2, or 6.4 mg/kg of SR144528 at a volume of 1–2 ml/kg. The initial dose of 3.2 mg/kg was selected based on prior behavioral investigations in male rats that suggest this dose does not antagonize CB1R (Craft et al., 2012). To determine if our observed outcomes were dose-dependent, a cohort administered 6.4 mg/kg of SR144528 was also included in our investigations.

### Testing Apparatus

Testing was conducted using eight HABITEST^®^ Operant Cages (24 × 30.5 × 29 cm; Coulbourn Instruments) placed in HABITEST^®^ Isolation Cubicles (Coulbourn Instruments, model: H10-24). The chambers were composed of two front and back aluminum walls, two clear acrylic side walls, a clear acrylic roof, and floor of stainless-steel rods (5 mm diameter) that were 1.5 cm apart from each other. Each isolation cubicle was equipped with an exhaust fan to provide background noise and ventilation. One of the aluminum sidewalls was outfitted with a food dispenser bordered by two retractable levers. Lever presses were automatically measured and entries into the food dispenser were recorded via a photocell using Graphic State software. The reinforcing stimulus was 45 mg banana flavored sucrose pellets.

### Autoshaping

The autoshaping protocol used in this study was modified from Khokhar and Todd (2018). Magazine training occurred on the first of 13 testing days. On the first day, rats were habituated to the apparatus. During this session, both levers were retracted, and one sucrose pellet was released approximately once every 30 s (± 30 s) for a 30 min session. No data was collected on this day.

Days 2–13 involved the rats learning to associate one of the two levers with reward delivery. Throughout the 60 min session, the conditioned stimulus lever (CS+) was presented 25 times, followed each time by the non-contingent delivery of a sucrose pellet, whereas the unconditioned stimulus lever (CS−) was presented 25 times without sucrose pellet delivery. Assignment of the left and right levers as the CS+ or CS− was counterbalanced in each group. The length of the inter-trial interval was randomized (60 ± 15 s). Lever presentation was pseudorandomized, with the same lever presented no more than two times in a row. Each lever was inserted into the chamber for 30 s. Food cup entries were only counted during CS+ lever presentations. Lever and food cup entry probabilities were calculated as the ratio of CS+ lever presentations with a lever press or food cup entry, respectively, divided by the total number of CS+ presentations in the session.

### Instrumental Learning

The 14-day instrumental learning protocol was adapted from Bouton et al. (2011). Two retractable levers were positioned in the chamber: one eliciting the presentation of a reward when pressed (CS+) and the other not paired with a reward delivery when pressed (CS−). The assignment of each lever as CS+ or CS− levers was pseudorandomized within each chamber such that the CS+ was assigned to the right lever and CS− assigned to the left lever in half of the chambers. All groups were counterbalanced for CS+ position throughout behavioral testing.

Rats underwent magazine training as described for the autoshaping protocol during days 1–2. During days 3–14, rats completed 32 min sessions of lever press training occurring on a variable interval 30 s reinforcement schedule. Both levers were inserted 2 min after session initiation and remained presented for the duration of the session. Approximately once every 30 s (± 30 s), the CS+ lever would enter a “working state” in which pressing the lever resulted in delivery of a sucrose pellet at the food dispenser. The lever would stay in this “working state” until pressed, after which the 30 s variable interval was reset.

### Data Collection and Statistical Analysis

The total number of CS− lever presses, CS+ lever presses and food dispenser entries were collected across experimental days. Results obtained from behavioral testing were evaluated with repeated-measures analyses of variance (RMANOVA) followed by Fisher’s *post hoc* testing when appropriate. Data were assessed using Greenhouse-Geisser correction when sphericity was violated followed by Fisher’s Least Significant Difference *post hoc* test where main effects were significant. Data analysis was performed using IBM SPSS Statistics 26. Results with a *p*-value < 0.05 were considered statistically significant.

## Results

### Adolescent SR144528 Treatment Alters Sign- and Goal-Tracking Behaviors in Adulthood

**FIGURE 1:**
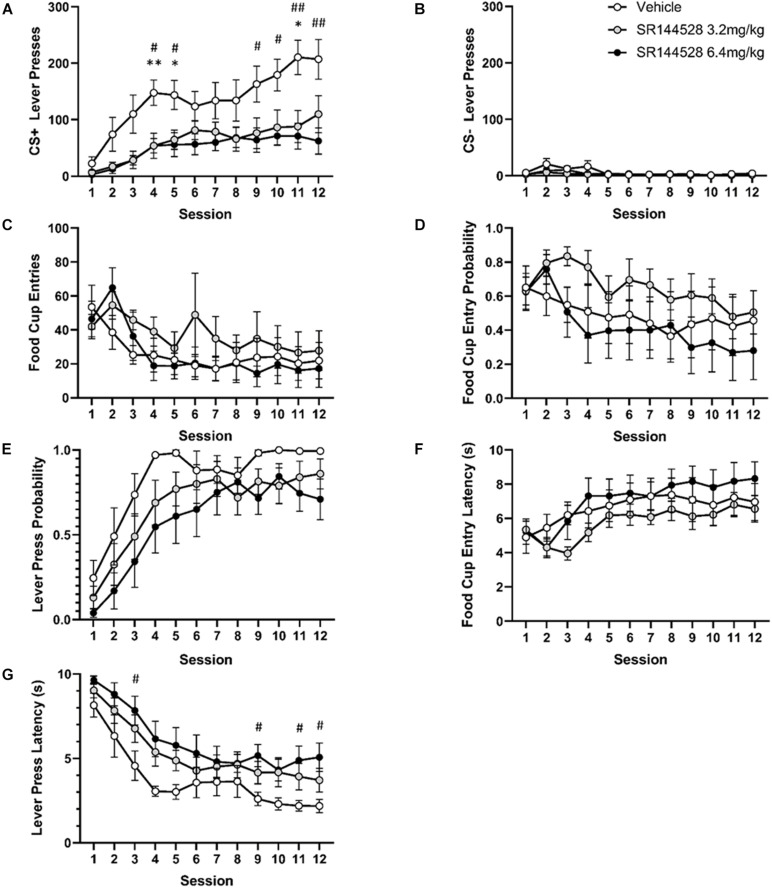
Antagonism/inverse agonism of CB2R during adolescence modulates adulthood autoshaping task performance. **(A)** Mean lever presses on the CS+ lever. **(B)** Mean lever presses on the CS– lever. **(C)** Probability of a lever press at least once during its presentation. **(D)** Probability of entering the food cup during CS+ lever presentation. **(E)** Mean latency to press the CS+ lever. **(F)** Mean latency to food cup entry. **(G)** Mean cumulative food cup entries during CS+ lever presentation. *p < 0.05, **p < 0.005 vehicle versus 3.2 mg/kg SR144528. ^#^*p* < 0.05, ^##^*p* < 0.005 vehicle vs 6.4 mg/kg SR144528. Data presented as mean ± SEM.

There was a significant main effect of adolescent SR144528 treatment [*F*_(2, 19)_ = 6.466, *p* = 0.0072], session [*F*_(11, 209)_ = 16.10, *p* < 0.0001], and a treatment by session interaction [*F*_(22, 209)_ = 1.598, *p* = 0.0489] on the number of CS+ lever pressing (Figure 1A). *Post hoc* analysis revealed that CS+ lever pressing was significantly greater in vehicle treated rats compared to rats treated with 3.2 mg/kg of SR144528 on sessions 4, 5, and 11 (*p* < 0.05). Similarly, the vehicle treated group pressed the CS+ lever more than the 6.4 mg/kg treated group on sessions 5, 6, and 9–12.

There was a significant main effect of session [*F*_(11, 209)_ = 3.749, *p* = 0.0180] and treatment [*F*_(2, 19)_ = 6.398, *p* = 0.0075] on CS− lever pressing, but no *post hoc* comparisons were significant (Figure 1B).

Food cup entries [*F*_(11, 209)_ = 4.714, *p* = 0.0036; Figure 1G] and food cup entry probability [*F*_(11, 209)_ = 2.990, *p* = 0.0313; Figure 1D] decreased across sessions. Lever press probability [*F*_(11, 209)_ = 28.66, *p* < 0.0001; Figure 1C] and food cup entry latency [*F*_(11, 209)_ = 6.949, *p* = 0.0002; Figure 1F] increased across sessions. There was no main effect of treatment, however, on food cup entries [*F*_(2, 19)_ = 0.6816, *p* = 0.5177; Figure 1G], probability [*F*_(2, 19)_ = 1.264, *p* = 0.3053; Figure 1D], or latency [*F*_(2, 19)_ = 1.175, *p* = 0.3302; Figure 1F], nor was there an interaction between session and treatment on food cup entries [*F*_(22, 209)_ = 0.7570, *p* = 0.7754; Figure 1G], probability [*F*_(22, 209)_ = 0.5403, *p* = 0.9551; Figure 1D], or latency [*F*_(22, 209)_ = 0.6401, *p* = 0.8916; Figure 1F].

There was a significant effect of session [*F*_(11, 209)_ = 36.24, *p* < 0.0001] and treatment [*F*_(2, 19)_ = 3.713, *p* = 0.0435] on latency to press the CS+ lever (Figure 1E). *Post hoc* multiple comparisons showed significantly lower latency to press in the vehicle group compared to the 6.4 mg/kg dose of SR144528 on sessions 3, 9, 11, and 12 (*p* < 0.05).

### Adolescent Exposure to SR144528 Does Not Affect Instrumental Learning in Adulthood

**FIGURE 2:**
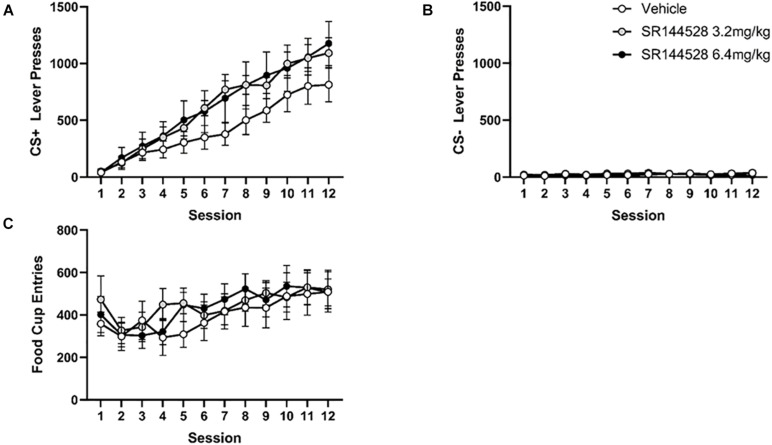
Antagonism/inverse agonism of CB2R during adolescence does not impact instrumental learning in adulthood. **(A)** Mean CS+ lever presses. **(B)** Mean CS– lever presses. **(C)** Mean cumulative food cup entries. Data presented as mean ± SEM.

There was overall increase in CS+ presses during acquisition with a main effect of session [*F*_(2, 21)_ = 1.204, *p* < 0.0001], but no effect of treatment on CS+ responding (Figure 2A).

Pressing of the CS− lever during learning acquisition decreased across sessions in all treatment groups (Figure 2B), though there was no significant main effects of session or treatment, nor any interactions.

Food dispenser entries during acquisition did not significantly differ across treatment groups (Figure 2C). There was a main effect of day [*F*_(4, 75)_ = 5.912, *p* = 0.01] such that the number of food cup entries increased over sessions.

## Discussion

We found that adolescent treatment with the CB2R antagonist/inverse agonist SR144528 reduced sign-tracking behavior in adulthood. Our results suggest that suppression of CB2R activity during adolescence impairs adulthood sign-tracking as evidenced by an attenuation of CS+ lever pressing and a shift toward the goal-tracking phenotype during autoshaping acquisition but did not impact instrumental learning. While there were no significant differences between the high and low dose treatment groups in this task, CS+ lever pressing behavior differed significantly between the high dose compared to the vehicle treated group on more days than the low dose group and was the only group to have significantly higher latency to press the CS+ lever compared to controls. We suspect that a potential ceiling effect on lever presses/probability, or floor effect on lever press latency may have contributed to our inability to see a dose-dependent relationship.

Localization of CB2R along the mesocorticolimbic pathway supports its role in reward circuitry and drug seeking behavior (Zhang et al., 2017). Acute CB2R activation has been shown to inhibit VTA DAergic neuronal firing and cocaine self-administration (Zhang et al., 2014), possibly through decreased DA released into the NAc (Zhang et al., 2014; Chase et al., 2015; Galaj and Xi, 2019). Similarly, it has recently been shown that CB2R-null mice also show elevated DA levels in the NAc in response to THC, further implicated CB2R as important mediators of mesolimbic DA signaling (Li et al., 2021). The decreased sign-tracking behavior seen following CB2R antagonism/inverse agonism in our study may, therefore, be due to a compensatory increase in NAc-VTA receptor sensitivity and/or expression following prolonged inhibition during adolescence (Zhang et al., 2014). It is well established that the NAc is integral to sign-tracking behavior (Chang et al., 2012), and lower accumbal DA is correlated with impaired cue-directed performance (Fitzpatrick and Morrow, 2016) whereas elevated DA neuron firing is apparent in rats that exhibit stronger reward-cue associations (Flagel et al., 2010; Chang et al., 2012; Chase et al., 2015). The discordant effects of adolescent CB2R pharmacological manipulation on Pavlovian and instrumental reward learning further support the modulation of DAergic signaling; flupenthixol (a DA receptor D1/D2 antagonist) blocks Pavlovian goal approach without impacting instrumental incentive learning (Wassum et al., 2011). However, studies also implicate CB2R activation may inhibit (Foster et al., 2016; Lopez-Ramirez et al., 2020) or stimulate (Lopez-Ramirez et al., 2020) DA release in the dorsal striatum that is contingent on non-cannabinoid receptors. As this region is relevant to both Pavlovian and instrumental learning through unique network recruitment (Chase et al., 2015), characterization of CB2R function across striatal networks and neuronal populations may reveal differential mechanisms within the dorsal striatum that are specific to sign- or goal-tracking behavior development.

Although we posit that long-term disruption of CB2R in mesolimbic circuits are underlying the observed alterations to sign-tracking behavior, other CB2R-related mechanisms may also be relevant to these findings. For instance, recent findings show that CB2R, but not CB1R, regulates physiological stress response to predator cues in rats such that their activity attenuates anxiety-like behavior following stress exposure (Ivy et al., 2020). Likewise, prolonged stress shifts rats from sign- to goal-directed behavior (Fitzpatrick et al., 2019). Prolonged inhibition of CB2R during adolescent development may therefore impair stress resilience in SR144528-treated rats, thus contributing to the shift from sign- to goal-directed reward learning mechanisms. This hypothesis is not mutually exclusive with the proposed involvement of CB2R expression in the VTA-NAc pathway; Ivy et al. (2020) detected increased *Cnr2* mRNA encoding CB2R mRNA in the PFC following predator stress. While they did not examine mRNA levels in the NAc or VTA, these increases were specific to the PFC and further implicate the mesocorticolimbic circuitry in CB2R’s role in cue-directed strategies.

While previous studies have assessed the impacts of adolescent exposure to agonists of both CB1 and CB2R on Pavlovian reward learning, our study adds specificity for CB2R in this process as well as explores of the impact of antagonism/inverse agonism. Adolescent exposure to the full CB1R/CB2R agonist WIN-55,212 increased goal-tracking in rats that also exhibited sign-tracking, producing an “intermediate” phenotype. Adolescent consumption of an edible form of THC, a partial agonist at both CB1R and CB2R, in male rats increased sign-tracking behavior (especially early in acquisition), while reducing goal-tracking behaviors in adulthood; these effects were not seen in female rats (Kruse et al., 2019). While the findings here cannot be directly compared due to methodological differences (e.g., length of conditioning, and different ages at exposure), our findings oppose the findings from these studies, consistent with the effects of SR144528. Our findings are also consistent with the effects of acute treatment with the CB1R antagonist rimonabant, where reductions in sign-tracking were observed (Bacharach et al., 2018). Based on consistent evidence between our study with another that used a non-specific cannabinoid receptor agonist THC (Kruse et al., 2019), alongside the observations of endogenous CB2R activity that may be related to learning (Fitzpatrick et al., 2019; Ivy et al., 2020), we suspect that adolescent treatment with a CB2R-specific agonist would shift Pavlovian reward learning toward sign-tracking and away from a goal-tracking phenotype.

Limitations of this study include the exclusive use of male rats (especially in light of the findings with edible THC highlighted above) and CB2R modulation only during early adolescence. In addition, SR144528 is both an antagonist and inverse agonist at CB2R, and has low affinity for CB1R (Rinaldi-Carmona et al., 1998; Portier et al., 1999). As the results of our study are consistent with the properties of SR144528 as an antagonist and an inverse agonist, we cannot conclude the pharmacological mechanism responsible for its influence on CB2R-mediated reward learning in adulthood. While a previous study suggests that SR144528 does not affect CB1R-mediated behaviors in male rats, its effects on these behaviors were inconsistent in females (Craft et al., 2012), and as such its use in females should be further validated before investigating sex-specific differences related to reward learning.

We report that inhibition of the CB2R during adolescence decreases sign-tracking behavior but does not affect goal-tracking behavior in adulthood. The present paper extends current literature on the impact of endocannabinoid system disturbances during adolescent development and adds to the expanding literature investigating cannabinoid receptors in the context of reward-related behaviors. Our paper provides new insights into adolescent receptor modulation of CB2R and provides evidence that CB2R may have a pivotal role in cue associative learning and reward motivation.

## Data Availability Statement

The raw data supporting the conclusions of this article will be made available by the authors, without undue reservation.

## Ethics Statement

The animal study was reviewed and approved by the University of Guelph Animal Care Committee.

## Author Contributions

HT, JF, and JK contributed to conception and design of the study. JF programmed the experiments. DE, BH, JF, HT, HK, and BJ performed the experiments. DE, BH, and JF performed statistical analysis. DE, BH, HT, and JF wrote sections of the manuscript. JK secured funding and supervised all students. All authors contributed to manuscript revision, read, and approved the submitted version.

## Conflict of Interest

The authors declare that the research was conducted in the absence of any commercial or financial relationships that could be construed as a potential conflict of interest.

## Publisher’s Note

All claims expressed in this article are solely those of the authors and do not necessarily represent those of their affiliated organizations, or those of the publisher, the editors and the reviewers. Any product that may be evaluated in this article, or claim that may be made by its manufacturer, is not guaranteed or endorsed by the publisher.

## Abbreviations

CB-R: Cannabinoid type—receptor
CS: Conditioned stimulus
DA: Dopamine
ECS: Endocannabinoid system
eCB: Endogenous cannabinoids
NAc: Nucleus accumbens
PCA: Pavlovian conditioning approach
PFC: Prefrontal cortex
PND: Postnatal day
VTA: Ventral tegmental area.
